# Psychometric properties of the WHOQOL-BREF in citizens from a disadvantaged neighborhood in Southern Sweden

**DOI:** 10.3389/fpsyg.2023.1118575

**Published:** 2023-04-11

**Authors:** Rathi Ramji, Margareta Rämgård, Anders Kottorp

**Affiliations:** Department of Care Science, Faculty of Health and Society, Malmö University, Malmö, Sweden

**Keywords:** Rasch analysis, quality of life, vulnerable population, migrants, health promotion, reliability

## Abstract

**Background:**

Citizens living in disadvantaged neighborhoods experience poorer health than the majority, and this inequality is a public health problem even in a welfare state such as Sweden. Numerous initiatives aimed at improving health and quality of life in these populations are being implemented and evaluated. Given that these populations are predominantly multicultural and multilingual, an instrument such as the WHOQOL-BREF, which is cross-culturally validated and available in multiple languages, may be appropriate. However, this cannot be ascertained since the psychometric properties of WHOQOL-BREF have never been assessed in the Swedish context. Thus, the current study aimed at assessing the psychometric properties of the WHOQOL-BREF questionnaire in citizens from a disadvantaged neighborhood in Southern Sweden.

**Methods:**

The respondents in this study were 103 citizens who participated in the health promotional activities of a Health promotional program and also responded to the 26-item, WHOQOL-BREF questionnaire as a part of an evaluation to assess the impact of the activities on the health-related quality of life of citizens. A Rasch model using WINSTEP 4.5.1 was used to assess the psychometric properties in this study.

**Results:**

Five of the 26 items, including pain and discomfort, dependence on medical substances, physical environment, social support, and negative feelings did not display acceptable goodness-of-fit to the Rasch model. On removing these items, the 21-item WHOQOL-BREF scale had an improved internal scale validity and person-separation reliability than the original 26-item version for this group of citizens from the neighborhood. When assessing the individual domains, three of the five items that were misfits on analyzing the full model also showed misfits in relation to two respective domains. When these items were removed, the internal scale validity of the domains also improved.

**Conclusion:**

WHOQOL-BREF seemed to be psychometrically inadequate when used in the original form due to internal scale validity problems, while the modified 21-item scale seemed better at measuring the health-related quality of life of citizens living in socially disadvantaged neighborhoods in Sweden. Omission of items shall be done but with caution. Alternatively, future studies may also consider rephrasing the items with misfits and further testing the instrument with larger samples exploring the associations between subsamples and specific item misfit responses.

## 1. Introduction

There is extensive evidence showing that populations living in socially disadvantaged neighborhoods experience poor health, have higher rates of illness and disability, and live shorter lives than the majority (De Snyder et al., 2011; Braveman, 2014; Cyril et al., 2015). This is in particular important to consider when measuring health among these citizens because they are often heterogeneous minorities who differ from the general population in terms of migration status, historical background, culture, and health practices (Alonge and Peters, 2015). These subgroups have special characteristics since they are not only different from the general population in the host country but also from their country of origin given the duration of their life in the host country. Thus, surveys used in these populations must be contextually validated to avoid bias owing to social circumstances (Antman et al., 2020). In addition, these surveys need to be easy to administer and must not impose a great burden on the respondent (Watson and Wooden, 2009). It is, therefore, important to monitor if target constructs (such as health) can be measured in a valid and precise way with the specific tool for this population, or if other approaches/tools are needed.

One of the major challenges in measuring health using conventional indicators such as mortality, morbidity, life expectancy, or biological functioning for these populations is that it fails to capture aspects of health beyond the presence of disease (Carr and Higginson, 2001). An alternative measure of health used in an attempt to address these challenges is the health-related quality of life, which is a subjective indicator of health and wellbeing. Quality-of-life is a comprehensive term used to describe a wide range of physical and psychosocial factors that may influence perceptions related to life. It is constructed based on human needs, more specifically on an individual’s perceived wellbeing, expectations, and phenomenological viewpoints (Ferrans et al., 2005). Health-related quality of life in specific concerns an individual’s subjective experiences of health and other external factors related to health, which may alter perceptions about life. The World Health Organization defines the health-related quality of life as a measure of an individual’s perception of their position in life in the context of their culture and value systems, and in relation to their goals, expectations, standards, and concerns (Ferrans, 2005).

Quality-of-life measures are increasingly used in many instances especially such as to test the effectiveness of interventions both as a supplement to biological measures or by themselves. Several quality of life surveys have been developed and validated globally. However, it is unclear whether these surveys take into consideration the cultural and contextual aspects of the respondent’s life, given that these factors also affect how individuals perceive health (Carr and Higginson, 2001). The WHOQOL-100 and WHOQOL-BREF, an abbreviated version of the former, were developed by the World Health Organization and are some of the commonly used quality of life surveys aside from the SF-36, which also measures health and wellbeing. The WHOQOL-BREF is, however, more popular since it is comparatively much broader and measures subjective domains taking into consideration the cultural and contextual aspects of the respondents (The WHOQOL Group, 1998a,b; Liang et al., 2008). It is convenient to administer and has been a preferred tool in cross-cultural studies given its availability in over 20 languages. A study among newly arrived Arabic migrants in detention centers in Sweden showed that the instrument had a high internal consistency (Puthoopparambil et al., 2015). Although the psychometric properties of the WHOQOL-BREF survey have been assessed in vulnerable contexts elsewhere in the world (Lin et al., 2016), it has not been tested among populations living in disadvantaged neighborhoods in Sweden.

Living in disadvantaged neighborhoods tends to have an impact on the perceptions of the population which may lead to psychological consequences altering their feelings related to health and wellbeing (Wrigley and Dawson, 2016). Thus, many mainstream objective indicators of quality of life and wellbeing may neither be valid nor reliable among disadvantaged groups as the components of these measures may not sufficiently reflect the values or priorities of these groups (Eikemo et al., 2017). Despite that WHOQOL-BREF has been cross-culturally validated and available in several languages (The WHOQOL Group, 1998a), its relevance or appropriateness is unclear for specific population subgroups particularly those that differ from the general population based on whom the instrument was originally developed. Although the language may be regarded as one of the factors to consider when administering surveys in a disadvantaged context, it must be noted that the social and cultural frameworks are frequently unique for certain subgroups, and some questions may be perceived differently or may even be meaningless in a given culture or context. Therefore, it is important to use contextually validated instruments to assess the quality of life of populations living in disadvantaged neighborhoods.

The current study aimed to assess the psychometric properties of the WHOQOL-BREF survey in a sample of citizens from a disadvantaged neighborhood in Southern Sweden.

## 2. Methods

### 2.1. Context

The current study was part of a larger program, Equal Health—Health Promotion Innovation in Collaboration, whose aim was to develop and evaluate health promotional initiatives using a participatory approach in a disadvantaged neighborhood in Sweden’s most multicultural city Malmö located in Southern Sweden (Ramji et al., 2020a,b). The health promotional program was based on a disadvantaged neighborhood with about 7,800 residents, of whom 75% are first- and second-generation migrants predominantly of Middle Eastern or East Asian origin. This neighborhood was one of the 15 neighborhoods that were enlisted as disadvantaged by the Swedish National Security Agency. Given the rise in health inequalities globally as well as in Sweden, there were several national and regional efforts to reduce inequalities and promote health, especially among populations in disadvantaged neighborhoods. Malmö commission was one of the first initiatives in Sweden much ahead of the national efforts and was based on the recommendations from the World Health Organizations’ report, Closing the Gap. The Malmö commission recommended that health promotional work in disadvantaged neighborhoods should be initiated over sectorial boundaries and in close collaboration with the local actors and communities. The Malmö municipality (Malmö stad) placed special focus on the neighborhood Lindängen, which was one of the disadvantaged areas listed by the National Security agency as there was a high rate of unemployment, low education, low income, and increased violence and criminality in this neighborhood. Furthermore, communities living in this neighborhood had poor health and low social mobility. Therefore, researchers from Malmö University together with the community members in Lindängen and local actors from public, private, and non-profit sectors established the Equal health program. The program had the aim of promoting health and wellbeing among citizens in the neighborhood.

### 2.2. Participants

Participants in this study (*n* = 103) were citizens from Lindängen who were involved with one of the four health promotional labs namely physical activity, women’s health, oral health, and mental health within the larger program. The activities in this project were collaborated by representatives from the neighborhood known as health promoters who among other things were facilitators or organizers of the different labs. These health promoters were instrumental in recruiting participants for the different activities using flyers and face-to-face interactions. Data for this study were gathered by these health promoters ahead of their participation in the health promotional labs. The questionnaires were collected in conjunction with a longitudinal study to evaluate the effect of the health promotional initiatives on the health-related quality of life of participants. The health promoters distributed the WHOQOL-BREF survey among participants in their respective labs and helped them in responding to the questions. However, the research team also educated the health promoters regarding methodological considerations during data collection to prevent the health promoters from influencing participant responses. They also reassured participants that the questionnaires were strictly anonymous and motivated participants to respond to all questions in an attempt to minimize missing responses.

### 2.3. Instrument

The WHOQOL-BREF is a generic, self-administered questionnaire with 26 items grouped into four domains each with specific facets. Of the 26 items, two of them namely perceptions of quality of life and health satisfaction, are labeled as global items. The remaining 24 items are divided into four domains representing different dimensions of quality of life including the physical health domain consisting of seven items, the psychological health domain with six items, the social relationships domain which includes three items, and finally the environmental health domain with eight items. The physical health domain included questions regarding the regular use of medicine and medical aids, activities in daily life, physical pain, fatigue levels, energy, and work capacity. While the psychological domain score included questions on self-reported stress, anxiety, positive and negative feelings, self-esteem, concentration, as well as body image and appearance. Questions on social support, personal relationships, and sexual activity were part of the social relationships domain. The environmental domain included contextual factors such as those related to the immediate environment including financial resources, safety, healthcare access, the physical environment, and transport (The WHOQOL Group, 1998b). Detailed information regarding the domains and the corresponding item numbers are provided in Table 1.

**Table 1:** Descriptive statistics of the individual items in the WHOQOL-BREF questionnaire.

Items	Domains	Mean (SD)
	Physical health domain	
17	How satisfied are you with your ability to perform your daily living activities?	3.28 (1.04)
4	How much do you need any medical treatment to function in your daily life?	2.84 (1.29)
10	Do you have enough energy for everyday life?	3.0 (0.94)
15	How well are you able to get around physically?	3.34 (1.03)
3	To what extent do you feel that physical pain prevents you from doing what you need to do?	2.88 (1.13)
16	How satisfied are you with your sleep?	2.90 (1.24)
18	How satisfied are you with your capacity for work	3.17 (1.09)
	Psychological health Domain	
11	Are you able to accept your bodily appearance?	3.45 (1.14)
26	How often do you have negative feelings such as blue mood, despair, anxiety or depression?	3.03 (1.08)
5	How much do you enjoy life?	2.94 (1.07)
19	How satisfied are you with yourself?	3.59 (1.15)
6	To what extent do you feel your life to be meaningful?	3.31 (0.99)
7	How well are you able to concentrate?	2.93 (1.11)
	Social relationships domain	
20	How satisfied are you with your personal relationships?	3.89 (0.87)
22	How satisfied are you with the support you get from your friends?	3.11 (1.21)
21	How satisfied are you with your sex life?	3.70 (0.98)
	Environmental health domain	
12	Have you enough money to meet your needs?	2.79 (1.02)
8	How safe do you feel in your daily life?	3.38 (1.07)
24	How satisfied are you with your access to health services?	3.22 (1.20)
23	How satisfied are you with the conditions of your living place?	3.52 (1.17)
13	How available to you is the information you need in your daily life?	3.16 (1.01)
14	To what extent do you have the opportunity for leisure activities?	2.87 (1.05)
9	How healthy is your physical environment?	3.41 (1.11)
25	How satisfied are you with your transport?	3.7 (0.94)
	Global items	
1	How would you rate your quality of life?	3.51 (0.96)
2	How satisfied are you with your health?	3.08 (1.18)

Responses for the different items are organized on a five-point Likert scale format where a higher score indicates better quality of life for most items except for three negatively phrased items, which were reversed for analysis. The World Health Organization recommends that the domain scores be initially calculated as a 26-item scale, and later be transformed to a 100-item scale. A higher score indicated a higher quality of life. The survey was reported to show good psychometric properties in both Swedish and Arabic versions according to previous studies based on other contexts (Skevington et al., 2004; al Sayah et al., 2013).

### 2.4. Analysis

The Rasch model was used to explore the psychometric properties of the WHOQOL-BREF survey, as well as, to understand if the domains constructed from multiple items conform to fundamental requirements of interval scales of measurement using the WINSTEPS computer software program version 4.5.1 (JM, 2015). A partial credit model was used as it was considered appropriate for ordered polytomous scales like that of WHOQOL-BREF where the responses for the different items have more than two categories (Masters, 1982). Although the rating scale model, which assumes equal distances between categories may also be used (Andrich, 1978), a partial credit model was assumed to better fit the data since the items in the different domains were not judged on the same kind of rating scales.

A Rasch model rather than the Classic Test Theory (CTT) was specifically chosen for this study since in the CTT approach, the latent traits for persons and items are often measured independently, and the scores are derived through summing responses across items. Second, the CTT values items with similar concepts equally and considers dissimilarities in scores between the two response scales to be uniform. Given that these scales may not be uniform in all data sets, CTT cannot be used to compare person-item relations. In contrast to CTTs, individual items can be analyzed using Rasch analysis to identify redundancies and item difficulty can also be assessed (Bradley, 2005). Although CTT has more frequently been used to test psychometric properties of the WHOQOL-BREF in other contexts, Rasch models may be advantageous particularly to assess the latent quality of life of participants in disadvantaged neighborhoods.

As a first step, item response-categories functioning was assessed. Responses were recoded where necessary to attain a better fit. Furthermore, local independence of items, item and person fit, unidimensionality, item separation reliability, and separation index was also assessed. Local independence among item residuals was assessed by examining the infit and outfit mean square (*MnSq*) to identify items as demonstrating misfit if their *MnSq* values were outside the interval < 0.7 to >1.3 (Smith, 1995). A larger accepted interval for fit was used for the participants (outside the interval < 0.6 to >1.4 with an associated *z-*value of ≥2 regarded as a misfit), with the consideration that up to 5% of the participants may fail to demonstrate acceptable goodness-of-fit without threatening evidence of person response validity (Linacre, 2002; Kottorp et al., 2003; Ann-Helen Patomella et al., 2006; Hällgren et al., 2011).

**Figure 1:**
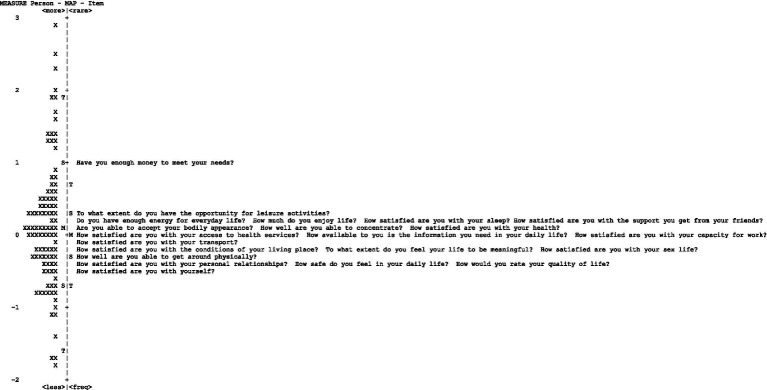
Person-item map.

Unidimensionality was assessed by examining the principal component analysis based on the criteria that ≥50% of the total variance is explained by the first component, and any additional component explains <5% of the variance associated with an eigenvalue of less than 2.0, of the remaining variance of residuals after removing first component. We also evaluated the Rasch model’s assumption of local independence among the items by monitoring the correlations between the item residuals, with a set criterion of a shared variance not larger than 25% (corresponding to a correlation coefficient larger than 0.5 between item residuals). Person reliability statistics of the Rasch model were also used to test the internal consistency among the items. The relationship between item difficulties and personal latent traits for each domain was assessed by examining the item-person map in the Rasch model analysis (Figure 1). The WHOQOL-BREF scale was expected to be able to distinguish at least three groups (indicating high, medium, and low levels of perceived health-related quality of life) with a separate index of at least 2.0 (Fisher, 1992). Finally, a differential test functioning (DTF) using standardized *z*-comparisons was performed to assess the consistency in measures across participants between the two versions of the WHOQOL-BREF (26-item and 21-item).

### 2.5. Ethical considerations

Ethical approval for the study was obtained from the Regional Ethical Review Board in Lund, Sweden (DNR 2018/591). All participants were verbally informed about the study goals, ahead of distributing the questionnaires. Participants were ensured that participation was voluntary nature, and they could choose to leave the study at any point in time. They were also informed that data collected in this study would be kept confidential and that only the research team would have access to it. In addition to the verbal information, participants also received a written copy of the above-mentioned information together with the contact information of the research team. Although one of the participants was below 18 years based on the Swedish law (2003:460) for ethical review of research involving humans, the parental concern may not be necessary if the research subjects themselves had the maturity to help realize what the research means for them and can provide their consent for participation. Given that this study did not include any biological test or measure and that the youth was sufficiently mature in accordance with the Swedish regulation the participant was individually informed about the research goals before obtaining his consent to participate. In addition, since the health promotional program as a whole focused on families living in a disadvantaged neighborhood the research team had contact with the 17-year-old youths’ mothers who were part of another activity. The parent was informed about her child’s engagement in this study and verbal consent was also obtained. Thus, all participants signed an informed consent ahead of participation.

## 3. Results

This study included 103 participants aged 17-80 years who were predominantly women. After performing initial control for missing responses to the survey, three female participants had missed a substantial portion of the surveys and therefore their responses were not included in the analysis. Participants in this study had origins from 24 different countries; however, nearly 80% of them were Arabic speaking. The demographic characteristics of the participants are presented in Tables 1, 2.

**Table 2:** Demographic characteristics of participants in this study.

Characteristics	Participants (*n* = 100)
*Age*	
Range (md)	17–82 (51)
*Gender*	
Women	90%
Men	10%
*Ethnicity*	
Middle Eastern	83%
Other	17%
*Educational Qualification*	
Less than 12 years	76%
Over 12 years	24%
*Language of response*	
Swedish	38%
Arabic	62%

Table 3 shows the results of each step of the Rasch analysis for both the whole WHOQOL-BREF instrument and the individual domains. Given that the four domains with 26 items all together are regarded as a measure of health-related quality of life, the Rasch analysis was initially performed for the whole 26-item scale. In addition, since each of the domains is also regarded as an individual construct, they were also analyzed separately.

**Table 3:** Psychometric properties of the WHOQOL-BREF scale.

Steps	Psychometric properties	Statistical approach and criterias	Original WHOQOL-BREF (26 items) results	Reduced WHOQOL-BREF (22 items) results	Reduced WHOQOL-BREF (21 items) results
1. Rating scale functioning: Does the rating scale function consistently across items?		Average measures for each step category on each item should advance monotonically *z*-values <2.0 in outfit mean square (*MnSq*) values for step category calibrations (Linacre, 2002)	Items 3,4, 11, 20.21,22, 23, 25, and 26 failed to meet the criteria	Item 16 failed to meet criteria.	All items met criteria
2. Internal scale validity: How well do the actual item responses match the expected responses from the Rasch model?		Item goodness-of-fit statistics (Smith, 1995). Infit *MnSq* values *m* < 0.7 to >1.3 and *z*-value <2.0	Four items (3,4, 23, and 26) failed to meet criteria.	Item 9 failed to meet the criteria.	All items met criteria
3. Is the scale unidimensional?		Principal component analysis of the residuals (Linacre, 2002). ≥ 50% of total variance explained by first component (quality of life). Any additional component explains <5% associated with an eigenvalue less than 2.0 of the remaining variance of residuals after removing first component.	First component explained 30% of the total variance. The second component explained 7% of the total variance with an eigen value of 2.6.	First component explained 36% of the total variance. The second component explained 6% of the total variance with an eigen value of 2.1.	First component explained 40% of the total variance. The second component explained 7% of the total variance.
4. Person-response validity: How well do the actual individual responses match the expected responses from the Rasch model?		Person goodness-of-fit infit. *MnSq* values *m* < 0.6 to >1.4 associated with a *z-*value <2.0 (Hällgren et al., 2011). ≤ 5% of sample fails to demonstrate acceptable goodness-of-fit values (Kottorp et al., 2003)	15% of the sample failed to demonstrate acceptable goodness of fit.	12% of the sample failed to demonstrate acceptable goodness of fit.	10% of the sample failed to demonstrate acceptable goodness of fit.
5. Person-separation reliability: Can the scale distinguish at least 3 distinct groups of perceived quality of life in the sample tested?		Person-separation index (Fisher, 1992) ≥ 2.0	2.62	3.05	3.05
8. Differential test functioning (DTF): How consistent are the scores from the modified 21-item and original 26-item WHOQOL-BREF		Standardized *z*-score differences+/−1.96 Pearson correlation *r* > 0.80 and *p* < 0.05			Three measures had *z*-scores <1.96 *r* = 0.973; *p* < 0.01

A total of 17 items met the criteria for acceptable rating scale functioning when analyzing the 26-item scale. Nine items displayed disordering and were recorded by collapsing the scale steps so that no more than two scale steps were reversed. For items 4, 11, 20, 22, and 25, scale step categories 1 and 2 were reversed. For items 3, 21, 23, and 26, scale step categories 2 and 3 were reversed, and for item 16, scale step categories 3 and 4 were reversed.

On examining the internal scale validity, any misfits were removed ahead of the next step, where a 22-item scale was analyzed. In this stage, one more item that displayed a misfit was also removed which resulted in a 21-item scale with no disordering or misfits. On assessing the item goodness fit of the 26-item version using a stepwise method, the items that failed to meet the criterion at a given step were removed and the step was repeated with the remaining items. Five items in total failed to demonstrate acceptable item goodness-of-fit, while the remaining 21 items had an acceptable item goodness-of-fit.

The principal component analysis indicated that both the 26-item and the 21-item versions failed to display ≥50% of the variance explained by the first dimension with an eigenvalue of 2.6 in the first contrast to more than 5% of the variance explained by the second dimension. Local independence among the items was confirmed, with no item residual correlations being higher than the set criterion or 0.5. The 21-item version demonstrated better person goodness-of-fit than the original 26-item WHOQOL-BREF; however, both versions failed to meet the criterion of ≤5% of the respondents demonstrating misfit.

Despite having fewer items, the 21-item version also demonstrated better person-separation reliability than the original 26-item version. Finally, the DTF analysis indicated that both versions of the scale (26-item and 21-item) generated similar measures for almost all respondents (97%). Only three respondents demonstrated *z*-values above the set criterion: all three demonstrated high health-related quality of life measures. In addition, both measures were highly correlated (*r* = 0.973; *p* < 0.05).

When Rasch models were used to assess the psychometric properties for the four domains of WHOQOL-BREF individually, some items within three of the domains displayed disordering in the rating scale function. Items 3, 4, and 7 in the physical health domain, items 11 and 26 in the psychological domain, and items 9 and 23 in the environmental health domain that showed disordering were recoded to the nearest response ahead of analysis in the second iteration.

Furthermore, some of the items in the physical health domain (two-item) and psychological health domain (one-item) failed to display acceptable item goodness-of-fit. On removing the misfit items in these two domains, the principal component analysis indicated ≥50% of the variance explained by the first dimension. Although the domain scales still failed the criterion set for the variance explained by the second dimension, and the person-separation index did not improve even after the misfit items were removed, the person-goodness fit improved for both domains (see Table 4).

**Table 4:** Psychometric properties of the physical and psychological domains of the WHOQOL-BREF scale.

Steps	Psychometric properties	Statistical approach and criterias	Original physical health domain (Seven-item) results	Reduced physical health domain (six-item) results	Reduced physical health domain (five-item) results	Original psychological health domain (six-item) results	Reduced psychological health domain (five items) results
1. Rating scale functioning: Does the rating scale function consistently across items?		Average measures for each step category on each item should advance monotonically *z*-values <2.0 in outfit mean square (*MnSq*) values for step category calibrations (Linacre, 2002)	Items 3, 4, and 17 failed to meet the criteria.	All items met the criteria.	All items met criteria.	Items 11 and 26 failed to meet the criteria.	All items met criteria.
2. Internal scale validity: How well do the actual item responses match the expected responses from the Rasch model?		Item goodness-of-fit statistics (Smith, 1995). Infit *MnSq* values *m* < 0.7 to >1.3 and *z*-value <2.0	Item 3 alone failed to meet criteria.	Item 4 failed to meet the criteria.	All items met criteria.	Item 26 failed to meet the criteria.	All items met criteria.
3. Is the scale unidimensional?		Principal Component analysis of the residuals (Linacre, 2002). ≥ 50% of total variance explained by first component (quality of life). Any additional component explains <5% of the remaining variance of residuals after removing first component.	First component explained 49% of the total variance. The second component explained 15% of the total variance.	First component explained 50% of the total variance. The second component explained 14% of the total variance.	First component explained 57% of the total variance. The second component explained 13% of the total variance.	First component explained 32% of the total variance. The second component explained 7% of the total variance.	First component explained 51% of the total variance. The second component explained 12% of the total variance.
4. Person-response validity: How well do the actual individual responses match the expected responses from the Rasch model?		Person goodness-of-fit infit MnSq values *m* < 0.6 to >1.4 associated with a *z*-value <2.0 (Kottorp et al., 2003), ≤ 5% of sample fails to demonstrate acceptable goodness-of-fit values (Kottorp et al., 2003).	10% of the sample failed to demonstrate acceptable goodness of fit.	9% of the sample failed to demonstrate acceptable goodness of fit.	7% of the sample failed to demonstrate acceptable goodness of fit.	16% of the sample failed to demonstrate acceptable goodness of fit.	9% of the sample failed to demonstrate acceptable goodness of fit.
5. Person-separation reliability: Can the scale distinguish at least 3 distinct groups of perceived quality of life in the sample tested?		Person-separation index ≥2.0 (Fisher, 1992).	1.7	1.6	1.9	1.5	1.6

No anomalies were exhibited on the assessed item goodness fit for the items within the Social Relationship domain. Although the items in the social relationship domain did not meet the criteria for variance explained by the first and second dimensions, the person separation index was also low (see Tables 5, 6).

**Table 5:** Psychometric properties of the social relationships and environmental health domain of the WHOQOL-BREF scale.

Steps	Psychometric properties	Statistical approach and criterias	Original Social Relationships Domain (3 items) results	Original Environmental Health Domain (8 items) results
1. Rating scale functioning: Does the rating scale function consistently across items?		Average measures for each step category on each item should advance monotonically *z*-values <2.0 in outfit mean square (*MnSq*) values for step category calibrations (Linacre, 2002).	All items met criteria.	Items 9 and 23 failed to meet the criteria.
2. Internal scale validity: How well do the actual item responses match the expected responses from the Rasch model?		Item goodness-of-fit statistics (Smith, 1995). Infit *MnSq* values *m* < 0.7 to >1.3 and *z*-value <2.0	All items met criteria.	All items met criteria.
3. Is the scale unidimensional?		Principal Component analysis of the residuals (Linacre, 2002). ≥ 50% of total variance explained by first component (quality of life) Any additional component explains <5% of the remaining variance of residuals after removing first component.	First component explained 48% of the total variance. The second component explained 29% of the total variance.	First component explained 41% of the total variance. The second component explained 14% of the total variance.
4. Person-response validity: How well do the actual individual responses match the expected responses from the Rasch model?		Person goodness-of-fit infit MnSq values *m* < 0.6 to >1.4 associated with a *z*-value <2.0 (Kottorp et al., 2003). 5% of sample fails to demonstrate acceptable goodness-of-fit values (Kottorp et al., 2003).	10% of the sample failed to demonstrate acceptable goodness of fit.	9% of the sample failed to demonstrate acceptable goodness of fit.
5. Person-separation reliability: Can the scale distinguish at least three distinct groups of perceived quality of life in the sample tested?		Person-separation index ≥2.0 (Fisher, 1992)	1.0	1.5

**Table 6:** Item difficulty, Rasch fit statistics for each item within the four domains in WHOQOL-BREF.

Item No.	Physical health domain text	Measure	SE	Infit MnSq (*z*-std)	Outfit MnSq (*z*-std)
16	How satisfied are you with your sleep?	0.27	0.11	1.02 (0.19)	1.03 (0.25)
4	*How much do you need any medical treatment to function in your daily life?*	0.23	0.11	1.41 (2.74)	1.40 (2.57)
3	*To what extent do you feel that physical pain prevents you from doing what you need to do?* *	0.19	0.12	1.09 (0.70)	1.05 (0.44)
10	Do you have enough energy for everyday life?	0.14	0.13	0.86 (−1.03)	0.86 (−1.00)
18	How satisfied are you with your capacity for work	−0.11	0.12	0.89 (−0.83)	0.98 (−0.11)
17	How satisfied are you with your ability to perform your daily living activities?	−0.31	0.12	0.87 (−1.01)	0.83 (−1.30)
15	How well are you able to get around physically?	−0.41	0.12	0.80 (−1.52)	0.80 (−1.59)
Item No.	Psychological health domain text	Measure	SE	Infit MnSq (z-std)	Outfit MnSq (z-std)
5	How much do you enjoy life?	0.16	0.12	0.83 (−1.25)	0.82 (−1.39)
7	How well are you able to concentrate?	0.04	0.12	0.89 (−0.78)	0.94 (−0.43)
26	*How often do you have negative feelings such as blue mood, despair, anxiety or depression?*	0.75	0.13	1.44 (3.01)	1.63 (3.80)
6	To what extent do you feel your life to be meaningful?	−0.32	0.13	0.73 (−2.07)	0.72 (−2.12)
11	Are you able to accept your bodily appearance?	0.02	0.13	1.16 (1.2)	1.33 (2.29)
19	How satisfied are you with yourself?	−0.65	0.12	0.93 (−0.46)	0.92 (−0.52)
Item No.	Social relationship domain text	Measure	SE	Infit MnSq (z-std)	Outfit MnSq (z-std)
22	How satisfied are you with the support you get from your friends?	0.39	0.13	0.88 (−0.76)	0.91 (−0.48)
21	How satisfied are you with your sex life?	−0.04	0.15	1.09 (0.66)	1.01 (0.10)
20	How satisfied are you with your personal relationships?	−0.35	0.16	1.00 (0.04)	0.89 (−0.67)
Item No.	Environmental health domain text	Measure	SE	Infit MnSq (z-std)	Outfit MnSq (z-std)
14	To what extent do you have the opportunity for leisure activities?	0.41	0.12	1.03 (0.29)	1.06 (0.51)
12	Have you enough money to meet your needs?	0.48	0.13	1.04 (0.36)	1.05 (0.40)
24	How satisfied are you with your access to health services?	0.10	0.11	1.01 (0.12)	1.06 (0.43)
13	How available to you is the information you need in your daily life?	0.05	0.12	0.94 (−0.38)	0.96 (−0.22)
25	How satisfied are you with your transport?	−0.04	0.13	0.92 (−0.57)	0.92 (−0.57)
23	How satisfied are you with the conditions of your living place?	−0.21	0.11	0.98 (−0.05)	0.96 (−0.18)
8	How safe do you feel in your daily life?	−0.30	0.12	0.82 (−1.38)	0.79 (−1.65)
9	How healthy is your physical environment?	−0.49	0.12	1.24 (1.73)	1.23 (1.64)

Items in italics do not demonstrate fit to the Rasch model in the first iteration of step 2.

* This item demonstrated misfit in the second iteration with six items [Infit MnSq (*z*-std) 1.41 (2.73); Outfit MnSq (*z*-std) 1.40 (2.69)].

## 4. Discussion

The current study shows that the WHOQOL-BREF seemed to have suitable psychometric properties for use among citizens from the socially disadvantaged neighborhood in Southern Sweden given that certain items are evaluated with caution. The precision/internal consistency of the instrument seems overall acceptable on the full 21-item version, but more questionable across the domains. Although originally developed for a broad group of respondents and having been tested in different parts of the world also in different languages, there were a few items in the instrument that failed to meet the criteria and fit into the quality of life measurement for this particular community. The item misfit could have been influenced by the semantics, e.g., the use of a dependence factor in the case of item 4 “*To what extent do you need medical treatment to be able to function in daily life*.” In addition, even complex phrasing of the various items, e.g., the question on home environment item 23 is worded as “*How satisfied are you with the conditions in which you live?*” which could denote much more than just their home environment and may also have led to different interpretations among the participants resulting in item misfit. Such questions need to be monitored more in-depth in future studies using cognitive interviews or think-aloud methodologies. Studies reporting findings using cognitive interviews of the WHOQOL-BREF in a socioculturally complex environment in Asia also show that phrasing and semantics influence responses (Zeldenryk et al., 2013).

On comparing the measures of the original 26-item version and the modified 21-item versions, the 21-item version displayed overall better internal scale validity for this particular group, and it may thus be preferred for valid measurements within such samples and contexts. It can also be argued that, due to the findings from the DTF analysis, an assessment done based on the valid 21-item version may still be comparable with previous studies that have used the 26-item original version. However, it is important to highlight that even though five items demonstrate misfits in this sample, and therefore should be treated with caution in generating measures of health-related quality of life for this sample, they may still contribute important information to describe and understand perceptions of quality of life among the participants. Future studies with larger samples should also monitor the associations between subgroups (e.g., gender, ethnicity, and language of response) and specific item responses in order to determine differential item functioning (DIF) in order to minimize potential unfairness in testing.

One major challenge with multidimensional instruments that are summarized into a unidimensional sum score is that we may have problems monitoring changes in relation to time or interventions if our measurements are “blurred.” Especially if the quality of life is viewed as a primary or secondary outcome in relation to health promotional interventions, we may under- or overestimate the potential changes if we are using the 26-item version of the WHOQOL-BREF, especially as interventions may address specific target variables/items. An analysis of health promotional outcomes for similar populations as in this study should therefore consider (a) using measures generated from the 21-item version of the WHOQOL-BREF, as this study provides evidence of multidimensionality with the 26-item version, (b) monitoring changes in both in relation to total sum score changes but also in relation to individual item score changes (e.g., by the use of differential item functioning (DIF) analysis; Petersson et al., 2008), and (c) taking into consideration the uncertainty of measures/scores generated from such tools. The latter is considered in a Rasch analysis output as each measurement is associated with an individual standard error (SE) that should be taken into account when estimating changes/effects.

While assessing the WHOQOL-BREF as an instrument measuring a unidimensional construct, five items (or questions) that failed to contribute to the quality of life model were not meeting the set criterion. Three of these five questions happened to be negatively phrased in the questionnaire. In addition, these questions also did not fit the individual domains they belonged to when being assessed domain-wise. Given that the group of participants was culturally bound migrants, these questions may have been sensitive to some of them, stigmatizing and thus they may have decided to provide more socially desirable responses (e.g., How satisfied are you with your sexual life?). A review study on the effect of negatively phrased questions also suggests that such a way of inquiry reverses the logic of the question to promote “disagreement” as a more socially acceptable response, causing confusion for the respondents (Colosi, 2005). The finding of this study regarding the removal of negatively phrased questions was similar to that observed in other instances where the same instrument was evaluated, including among the Taiwanese population dependent on heroin, the Iranian adult population, Taiwanese lung cancer patients, and Arabic speaking populations from Saudi Arabia (Ohaeri and Awadalla, 2009; Usefy et al., 2010; Chang et al., 2014; Lin et al., 2017). However, the Taiwanese study only assessed the psychometric properties of the different domains in special populations with heroin addiction and did not assess if these domains together contributed to the primary construct of quality of life (Chang et al., 2014). The Iranian study, in contrast, differed from our study in that they aimed at identifying differences in the use of the instrument between healthy and unhealthy samples of the general population (Usefy et al., 2010). The study from Saudi Arabia is of particular interest in relation to this study since participants in the current study were predominantly of Middle Eastern origin and responded to the questionnaire in Arabic. One of the important findings in the Saudi Arabian study aside from those that were similar to our current study was that the social relationship domain had serious misfit issues owing to high percentages of missing data for questions participants later related to as sensitive such as sexual activity (Ohaeri and Awadalla, 2009). In contrast to this, the current study did not have missing responses since the participants were assisted by community representatives namely health promoters while they responded to the questions. These health promoters constantly encouraged the participants when responding to the questions and were available to clarify any concerns participants had while they responded to the questionnaire.

## 5. Limitations

The main limitations of this study include the inclusion of participants based on a convenient sampling procedure, with participants living in disadvantaged neighborhoods and volunteering to participate in health-promoting activities. There were not matched for attaining demographic diversity but were regarded as representative of the entire community. The population sampled was predominantly women, and this may also be considered a limitation since the results may not be completely generalizable. However, this study was part of a larger program that mainly included women migrants who were frequently excluded from larger health promotional initiatives (Lindsjö et al., 2021; Avery et al., 2022). Hence, they can be regarded as representative of the cohort population under study.

Recoding some of the response categories in our analysis, changing the total achievable domain scores and converting the scores to match the WHOQOL-100 may not be according to the standard rules provided by the World Health Organization guidelines. However, specific reference ranges that indicate better or poor quality of life do not guide the final scores. Thus, the consequences of restructuring this scale may not largely affect the final interpretation of the scores. Consequently, it is not possible to provide a straightforward ordinal-to-interval scores’ conversion table. However, as previous research suggests a reconstructed WHOQOL-BREF instrument score should be evaluated using non-parametric statistics (Chang et al., 2014).

## 6. Conclusion

The current study has elucidated that an item-reduced WHOQOL-BREF generates valid measures of health-related quality of life from citizens living in disadvantaged neighborhoods compared to the original 26-item version. However, it may not be fully desirable to directly remove the negatively phrased items that caused internal validity problems. Future studies must consider alternative ways to present such questions for achieving improved participant responses and reevaluating the instrument. If the instrument is applied in its original form, the results are to be interpreted with caution and possibly complemented by other qualitative methods of evaluation.

## Data availability statement

The raw data supporting the conclusions of this article will be made available by the authors, without undue reservation.

## Ethics statement

The studies involving human participants were reviewed and approved by The Regional Ethical Committee in Lund (DNR 2018/591). The participants provided their written informed consent to participate in this study.

## Author contributions

AK, MR, and RR participated in the design of the study. RR and MR collected data for this study together with the health promoters. RR and AK analyzed the data. RR wrote the first version of the manuscript which was later revised by AK and MR. All authors contributed to the article and approved the submitted version.

## Funding

The study was part of a larger project financed by VINNOVA (DNR 2016-00421 and 2017-01272). The VINNOVA funding was primarily toward the establishment of a health promotion program and did not support research conducted within this program.

## Conflict of interest

The authors declare that the research was conducted in the absence of any commercial or financial relationships that could be construed as a potential conflict of interest.

## Publisher’s note

All claims expressed in this article are solely those of the authors and do not necessarily represent those of their affiliated organizations, or those of the publisher, the editors and the reviewers. Any product that may be evaluated in this article, or claim that may be made by its manufacturer, is not guaranteed or endorsed by the publisher.
